# Improvement of the Physicochemical Limitations of Rhapontigenin, a Cytotoxic Analogue of Resveratrol against Colon Cancer

**DOI:** 10.3390/biom13081270

**Published:** 2023-08-20

**Authors:** Silvia Navarro-Orcajada, Francisco José Vidal-Sánchez, Irene Conesa, Adrián Matencio, José Manuel López-Nicolás

**Affiliations:** 1 Departamento de Bioquímica y Biología Molecular-A, Facultad de Biología, Universidad de Murcia—Regional Campus of International Excellence “Campus Mare Nostrum”, E-30100 Murcia, Spain; 2 Dipartimento di Chimica, Università di Torino, via P. Giuria 7, 10125 Turin, Italy

**Keywords:** stilbene, cancer, food, solubility, stability, encapsulation

## Abstract

It has been argued that methoxylated stilbenes are better candidates for oral administration than hydroxylated stilbenes, including resveratrol, as they share many biological activities but have better bioavailability. By contrast, they have a disadvantage to consider, i.e., their lower hydrophilic character that leads to precipitation issues in the final product. In this work, we analysed and compared the growth inhibition of colorectal cancer cells of the methoxylated stilbene rhapontigenin and some analogues and overcame potential problems in the development of fortified products by designing inclusion complexes. Among several cyclodextrins, we found the one that best fit the molecule by physicochemical and bioinformatics assays. The stoichiometry and the encapsulation constants with natural and modified cyclodextrins were determined by fluorescence spectroscopy. The most promising complexes were analysed at different temperature and pH conditions, determining the thermodynamic parameters, to discover the optimal conditions for the preparation and storage of the products. The results showed that rhapontigenin solubility and stability were significantly improved, achieving a sevenfold increase in water solubility and maintaining more than 73% of the stilbene after three months. These findings could be of great interest for industries that aim to deliver novel bioactive compounds with higher solubility and lower degradation.

## 1. Introduction

Stilbenes are a family of phenolic compounds produced by different plant species such as grapes or peanuts in response to biotic and abiotic stress conditions, acting as phytoalexins. These secondary metabolites share a 1,2-diphenylethylene structure, but the aromatic rings are often substituted with a different number and type of chemical groups (hydroxyl, methoxy, glucosyl, etc.) [1].

Stilbenes are widely studied as bioactive compounds that can be used for the prevention and treatment of human disorders. These compounds have been reported as having many interesting bioactive properties, such as their antioxidant, anti-obesity, cardioprotective, neuroprotective, and anti-cancer activity, among others [1,2,3]. The most studied stilbene is resveratrol (*trans*-3,4′,5-trihydroxystilbene), which has been tested in many different in vitro studies. However, information on its in vivo properties remains scarce due to its low bioavailability. In humans, resveratrol bioavailability has been reported to be less than 1% by oral administration [4]. Despite this disappointing fact, there are some stilbene analogues with enhanced activities or different physicochemical properties due to the nature of their substituents [1].

Rhapontigenin (RHA, *trans*-3,3′,5-trihydroxy-4′-methoxystilbene) is a natural methoxylated stilbene biosynthesized by certain plant species such as *Rheum undulatum*, from the *Polygonaceae* family. Recent studies demonstrated that it has numerous bioactivities such as antioxidant, antithrombotic, hypoglycaemic, antihyperlipidemic, antimicrobial, anticancer, cardioprotective, and neuroprotective effects [5,6,7,8,9,10]. These effects make RHA a very interesting molecule, as the industry is constantly looking for new bioactive ingredients for the development of more effective products.

Compared to resveratrol, the more hydrophobic structure of RHA could be of great interest for the bioavailability of the molecule [1]. However, it could also have a negative effect in the solubility of the molecule, which could pose a challenge in product design. Formulations that include cyclodextrins (CDs) have been proved to improve the solubility of several bioactive compounds, in addition to protecting the molecule from degradation, evaporation, and oxidation [11,12,13,14].

CDs are cyclic oligosaccharides formed by different units of glucose molecules joint by α-(1→4) links. These molecules have a hydrophilic external surface and a hydrophobic internal cavity that can encapsulate molecules that are poorly soluble in water, improving their aqueous solubility [15,16]. There are three natural CDs depending on the number of glucose units forming the ring: α-CD (six units), β-CD (seven units), and γ-CD (eight units). These three natural CDs have been approved as food additives (E-457, E-459, and E-458, respectively), but there are also modified CDs like methyl-β-CD (M-β-CD) and hydroxypropyl-β-CD (HP-β-CD) currently used in therapy [17].

To enable the potential applications of RHA in the medical industry, this is the first study to compare the antiproliferative activity against colorectal cancer of RHA and other better-known analogues and to analyse its molecular encapsulation in CDs, determining the stoichiometry of the inclusion complexes, the most suitable CD to use, the influence of temperature and pH on the complexes, and the thermodynamic parameters. Furthermore, these results were complemented with bioinformatics to predict the 3D interactions and affinity. Finally, a comparative evaluation was carried out to test whether the formulation can improve the solubility and stability of this promising ingredient. 

## 2. Material and Methods

### 2.1. Materials

RHA was obtained from TCI (Zwijndrecht, Belgium). Cyclodextrins α-CD, β-CD, and γ-CD were purchased from Sigma Aldrich (Madrid, Spain), while methyl-β-cyclodextrin (M-β-CD, DS = 5.4) and 2-hydroxypropyl-β-cyclodextrin (HP-β-CD, DS = 5) were from Carbosynth (Berkshire, UK).

### 2.2. Methods

#### 2.2.1. Cell Line and Culture Conditions

Caco-2 cell line (human colorectal cancer) was obtained from the American Type Culture Collection (ATCC, Rockville, MD, USA) and was cultured according to ATCC guidelines. Cells were grown in complete EMEM culture medium with 10% (*v*/*v*) foetal bovine serum and 0.1 mg/mL penicillin/streptomycin, in an incubator at 37 °C, 5% CO_2_, and 85% relative humidity.

#### 2.2.2. Cell Viability Evaluation

Colorectal cancer cell (passage 34–37) density was determined with trypan blue in a TC10™ automated cell counter (Bio-Rad, Madrid, Spain), and 10,000 cells per cm^2^ were seeded in 96-well plates. The peripheral wells were filled with sterile water in order to avoid evaporation. Cells were incubated for 48 h before changing the medium to fresh medium supplemented with the treatments. The stilbenes’ stock solutions were solubilised in dimethyl sulfoxide (final DMSO 0.33%) and filter sterilised (0.2 µm) before their addition to the culture medium. The treatments had a final concentration of rhapontigenin, resveratrol, and piceatannol of 25, 50, and 100 µM and were incubated 48 h. Controls containing 0.33% DMSO were also prepared.

Cell viability was measured by neutral red method [18]. First, a 4 mg/mL neutral red stock solution was prepared in PBS and stored at room temperature in darkness. This solution was diluted 1:100 in complete growth medium before being used. Cells were washed with PBS and incubated with the diluted solution of neutral red. After 2 h, the cells were washed again, air dried, and an unstaining solution (ethanol + water + acetic acid (1:1:0.02)) was added. Cells were subjected to gently oscillation for 10 min before reading the absorbance at 540 nm and 690 nm (background absorbance) on an FLUOstar Omega plate reader (BMG Labtech, Ortenberg, Germany). A blank with no cells was also considered. Final results were expressed as the % Cell Viability according to Equation (1):

(1) $$\%\;Cell\;Viability=\frac{A_{540-690}\;(Treated\;Cells)}{A_{540-690\;}(Untreated\;Cells)}\times 100$$

#### 2.2.3. Fluorescence Measurements

Maximum emission and excitation wavelength were determined on a Shimadzu RF-6000 spectrofluorometer with thermostatically controlled cells and emission and excitation bandwidths set at 5 nm. The formation of the inclusion complexes was controlled on a Kontron SFM-25 spectrofluorometer (Kontron, Zurich, Switzerland) with thermostatically controlled cells, a xenon lamp source, 2 nm quartz cells, and setting emission and excitation bandwidths at 2 nm. Inclusion complexes were prepared with CD concentrations from 0 to 10 mM and RHA at 25 μM in 0.1 sodium phosphate buffer pH 7. The effect of pH was evaluated using 0.1 M sodium acetate buffer pH 3, 0.1 sodium phosphate buffer pH 5, 0.1 sodium phosphate buffer pH 7, and 0.1 sodium borate buffer pH 9. The effect of temperature was evaluated by incubating the solutions for thirty minutes at 5, 15, 25, or 35 °C.

#### 2.2.4. Mathematical Model to Calculate the Stoichiometry and Encapsulation Constants

The stoichiometry of the inclusion complexes was determined by considering that the guest molecule can be encapsulated in one host molecule (i.e., 1:1 stoichiometry) or two host molecules (i.e., 1:2 stoichiometry). Equations (2) and (3) describe the equilibrium of both models:

(2) RHA+CD ⇄ RHA−CD

(3) RHA+2 CD ⇄ CD−RHA−CD

Using the Benesi–Hildebrand method [19], the encapsulation constants (*K_F_*) (Equation (4)) were calculated with the relative fluorescence signals with and without cyclodextrins (Equation (5)). The higher the encapsulation constant was, the more stable the inclusion complex.

(4) $$K_{F}=\frac{\left[RHA-{CD}_{x}\right]}{\left[RHA\right]\cdot {\left[CD\right]}^{x}}$$

(5) $$\frac{1}{F-F_{0}}=\frac{1}{\left(F_{\infty }-F_{0}\right)\cdot K_{F}\cdot {\left[CD\right]}^{x}}+\frac{1}{F_{\infty }-F_{0}}$$

where [*RHA − CD_x_*], [*RHA*], and [*CD*] are the equilibrium concentrations, *F*_0_ is the basal fluorescence intensity of *RHA*, *F*_∞_ is the fluorescence intensity when all *RHA* is encapsulated in *CDs*, *F* is the observed fluorescence intensity of *RHA* at each *CD* concentration, and *x* is the stoichiometry coefficient (equal to 1 in 1:1 model and 2 in 1:2 model). 

#### 2.2.5. Molecular Docking

The natural α-CD and β-CD were obtained from a crystal from the Protein Data Bank (PDB ID: 2XFY and 1Z0N), while γ-CD was extracted from the London South Bank University website. The modified HP-β-CD and M-β-CD were created by adding hydroxypropyl or methyl groups to the β-CD structure. The topology of the modified CDs was obtained using PRODRG with default parameters, while default topology was used for the remaining molecule. The RHA was obtained from the PubChem database (CID 5320954) (NCBI, Rockville, MD, USA). Input files for molecular docking were generated using Autodock tools (version 1.5.6) with default parameters and charges. Molecular docking was performed with Autodock Vina [20] using default parameters and considering the flexible atoms of the cyclodextrins. A seed of 5000 was set at the beginning of the run. Final images were prepared in PyMOL (Molecular Graphics System, version 1.3, Schrödinger, Mannheim, Germany) with default parameters to show hydrogen bonds.

#### 2.2.6. Determination of the Thermodynamic Parameters of the Inclusion Complexes

The thermodynamic parameters of the most stable RHA complexes with CD were calculated with Equations (6) and (7).

(6) $$\ln K_{F}=\frac{-∆H{}^{\circ}}{RT}+\frac{∆S{}^{\circ}}{R}\;$$

(7) ∆G°=∆H°−T·∆S° 

where Δ*H*° is the standard enthalpy change, *R* is the gas constant, *T* is the temperature, Δ*S*° is standard entropy change, and Δ*G*° is the Gibbs free energy of complex formation. 

#### 2.2.7. Determination of Aqueous Solubility

Saturated solutions of RHA at 1 mg/mL in water were exposed to different concentrations of HP-β-CD (0, 1, 5 and 10 mM) and incubated 10 min in the dark at 25 °C. The solutions were then centrifuged and the supernatant diluted 1:100 in ethanol to measure absorbance at 326 nm in a Jasco V-630 Spectrophotometer with Thorlabs cuvettes CV10Q1400. The remaining concentration of soluble RHA was calculated by determining the molar attenuation coefficient (ε) of RHA. For this purpose, the absorbance between 200 and 600 nm of ethanolic solutions of RHA at increasing concentration was recorded, and the Lambert–Beer law was used.

#### 2.2.8. Stability Test of Inclusion Complexes

Since most foods, drugs, and cosmetics are stored at room temperature, we tested the stability of this bioactive ingredient in free form and encapsulated in HP-β-CD. Formulations of RHA were prepared at 25 μM with and without HP-β-CD (1, 5, and 10 mM) and kept at pH 7, 25 °C, and darkness. Stability was assessed by determining absorbance at 326 nm every two week for 3 months in a Jasco V-630 Spectrophotometer with Thorlabs cuvettes CV10Q1400 and using a blank with buffer and ethanol at the same concentration as the samples. Results were expressed as the percentage of the initial amount.

#### 2.2.9. Data Analysis

All experiments were performed in triplicate. Regressions were performed using Sigma-Plot (version 10.0.0.54). A *t*-test was performed using Rstudio (version 0.99.878) with a significance of *p* < 0.05. 

## 3. Results and Discussion

### 3.1. Cytotoxicity in Human Colorectal Cancer Cells Treated with RHA and Stilbene Analogues

**Figure 1:**
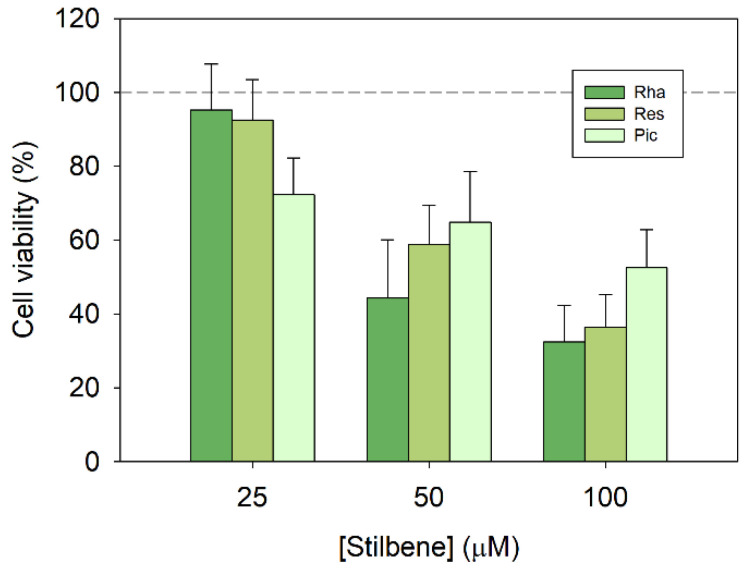
Cytotoxicity of rhapontigenin (Rha) and its main analogues, resveratrol (Res) and piceatannol (Pic), on human colorectal cancer cell line Caco-2 after 48 h treatment.

After a 48 h treatment, concentrations of stilbenes above 25 µM decreased Caco-2 cells’ viability in a dose-dependent manner (Figure 1). At the lowest concentration, piceatannol was the only stilbene to show cytotoxic effects, while RHA and resveratrol showed no difference to control cells. Despite this, RHA and resveratrol were able to achieve greater cancer cell death with increasing concentration. In fact, the highest concentration tested (100 µM) showed that the cytotoxicity of piceatannol was significantly lower than that of RHA, leaving a cancer cell survival of 53%, while RHA and resveratrol gave 32% and 36%, respectively. Meanwhile, the cytotoxicity of RHA at 50 µM was slightly better than that of resveratrol and piceatannol, but still not a significant difference.

Despite being structurally similar, these stilbenes show differences in their cytotoxic activity. Although piceatannol is the most effective at low concentration, it apparently plateaus with increasing concentration, probably because its higher number of free hydroxyl groups makes it more reactive [1]. By contrast, resveratrol has a more pronounced and linear increase in activity over the range of concentrations tested, and rhapontigenin shows the greatest increase in activity between 25 and 50 µM, reaching the highest percentage of cell death at 100 µM. Overall, these results indicate that RHA could have a similar or even slightly better antiproliferative activity against human colorectal cancer cells than other more common stilbenes, such as resveratrol and piceatannol, respectively. RHA also showed inhibition of cell growth in prostate and liver cancer cell lines [8,21]. However, the effects that we have observed in Caco-2 colorectal cancer cells in this study were markedly better, which encourages the study of the activity and applications of this stilbene.

### 3.2. Calculation of Stoichiometry and Selection of the Most Suitable CD to Complex RHA by Fluorescence Spectroscopy

**Figure 2:**
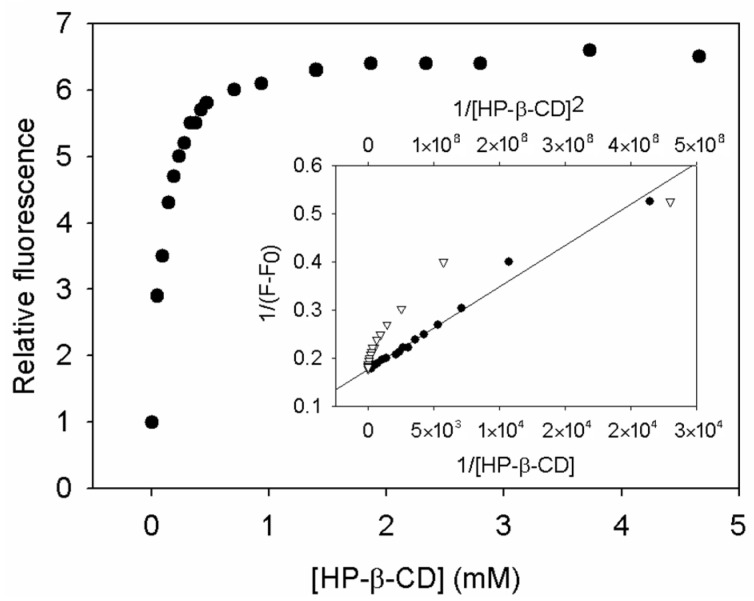
Complexation curve of RHA with HP-β-CD (25 °C pH 7). Inset: Benesi–Hildebrand fitting for a (●) 1:1 stoichiometry and (▽) 1:2 stoichiometry.

Encapsulation curves were obtained for each CD to determine the stoichiometry and encapsulation constants of the inclusion complexes (Figure 2). Except γ-CD, which could not fit any model, all CDs gave R^2^ > 0.942 for the 1:1 model, but R^2^ < 0.896 for the 1:2 model (Table 1). The higher correlation coefficients in the first model reveal that one RHA molecule interacts with one molecule of CD, i.e., the complexation follows a 1:1 stoichiometry. This result correlates well with the encapsulation of other stilbene analogues, such as piceatannol, resveratrol, gnetol, and oxyresveratrol [1].

RHA encapsulation constants were determined with each CD using Equation (5) (Table 1). The modified HP-β-CD gave the highest value (*K_F_* = 10,308.89 ± 515.44 M^−1^), which means that the cavity of this CD was the best to accommodate the stilbene. Figure 2 shows the complexation curve and the Benesi–Hildebrand fitting of the RHA complexes with HP-β-CD. Apart from the better correlation with the 1:1 stoichiometry, it can be seen that the fluorescence signal reaches a plateau at a very low concentration of 0.5 mM.

Among the other CDs, M-β-CD (*K_F_* = 5189.11 ± 259.45 M^−1^) gave an encapsulation constant half that of HP-β-CD, while β-CD (*K_F_* = 2990.11 ± 149.51 M^−1^) and α-CD (*K_F_* = 520.32 ± 26.02 M^−1^) constants were more than three times and almost twenty times lower than HP-β-CD, respectively. The encapsulation constant with γ-CD could not be determined due to the small increase in the fluorescence signal after CD addition. 

These values are in the range of that previously obtained with the analogue piceatannol [22], although the order of CD preference changes slightly (HP-β-CD > β-CD > M-β-CD > γ-CD > α-CD). In any case, both stilbenes showed a higher encapsulation efficiency with natural and modified β-CDs, especially with HP-β-CD.

### 3.3. Computational Selection of the Most Suitable CD to Complex RHA

**Figure 3:**
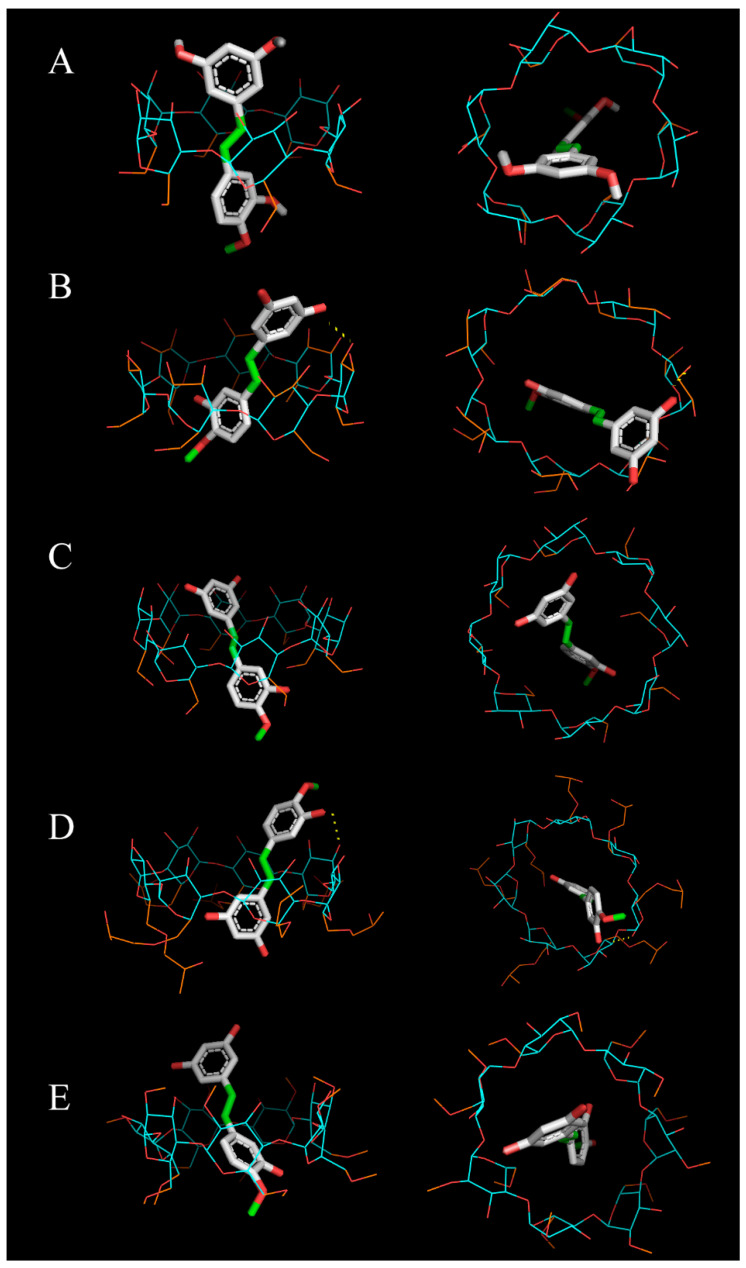
3D modelling of RHA complexes with (**A**) α-CD, (**B**) β-CD, (**C**) γ-CD, (**D**) HP-β-CD, and (**E**) M-β-CD. The flexible and non-flexible CD atoms are coloured in orange and blue, respectively, while the hydrogen bonds are yellow dotted lines.

The Autodock Vina outputs for each inclusion complex agreed with the experimental results shown above. The docking scores are proportional to the Gibbs free energy, and, therefore, lower scores are desirable to develop more stable complexes. The HP-β-CD score was the lowest, meaning that this CD was the most suitable for encapsulating RHA among the studied CDs (Table 1). Notably, this docking was the only one in which RHA enters the internal cavity with the methoxy group on the secondary face of the CD (Figure 3D). It is suggested that the orientation of the guest molecule in the host cavity may play a relevant role in the affinity of the complex, with this assembly with the methoxylated ring near the secondary face of the CD being the most favourable. In addition, this molecular docking showed hydrogen bonds between RHA and HP-β-CD, which enhance encapsulation stability.

Although M-β-CD and β-CD scores were equal (Table 1), the latter 3D modelling showed hydrogen bonds (Figure 3B), a key interaction between molecules that stabilises the inclusion complex. As in the fluorimetric results, α-CD and γ-CD show the worse scores. Although a score has been defined bioinformatically, it has previously been shown experimentally that γ-CD did not have sufficient affinity for RHA.

### 3.4. Effect of pH on RHA Inclusion Complexes with HP-β-CD

**Figure 4:**
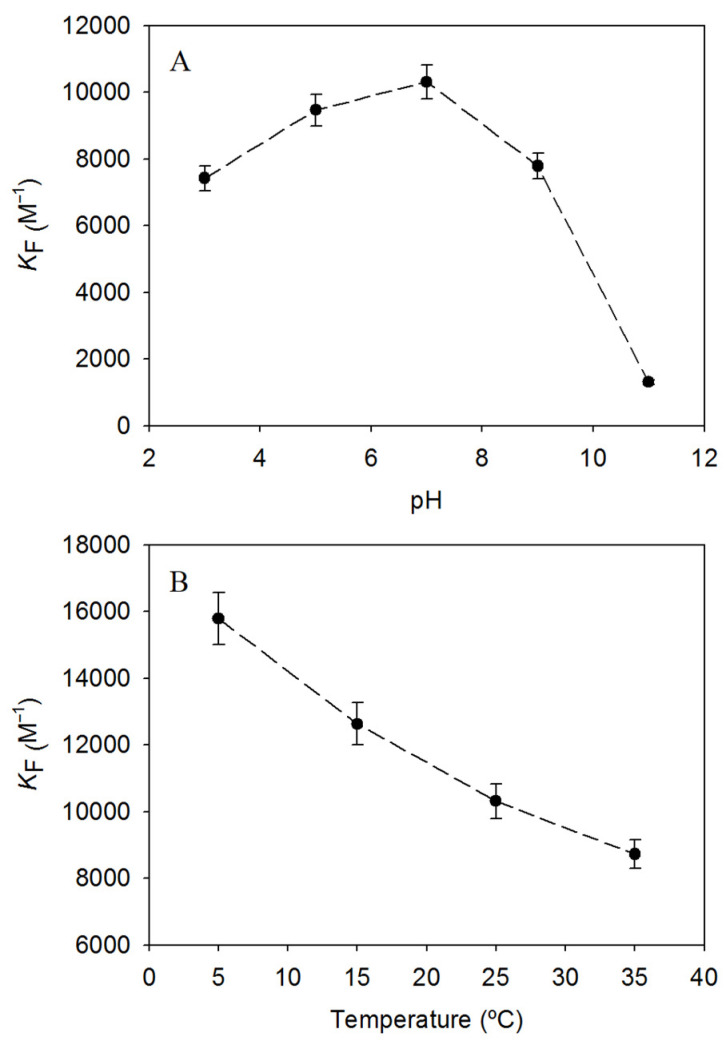
Influence of (**A**) pH and (**B**) temperature on the encapsulation constants of RHA with HP-β-CD.

The inclusion complexes with CDs that gave the best encapsulation were exposed to variations in the pH of the medium to assess whether the complex was affected by this parameter. Indeed, the encapsulation constants changed with varying pH (Figure 4A). The inclusion complexes were more stable at pH 7, which was observed in a higher encapsulation constant. While changes at acidic pH caused a small loss of stability (from 8% at pH 5 to 28% at pH 3), changes at basic pH trigger a sharp decrease in the encapsulation constant, reaching *K_F_* = 1305.68 ± 65.28 M^−1^ at pH 11, i.e., a decrease of 87% compared to *K_F_* at pH 7. The higher constant at neutral pH is an attractive result, as the inclusion complexes would be more stable under physiological conditions.

This region where the encapsulation constants drop drastically has been previously associated with a change in the protonation state of the molecule [1,22]. Therefore, RHA dissociation constants could be close to basic pH, and to develop a stable formulation of RHA with CDs, it would be better to use neutral pH.

### 3.5. Effect of Temperature on RHA Inclusion Complexes with HP-β-CD and Determination of Thermodynamic Parameters

Encapsulation constants of RHA with HP-β-CD and temperature were inversely related with a very good coefficient R^2^ = 0.98 (Figure 4B). The highest constant was found at 5 °C *K_F_* = 15,785.24 ± 789.26 M^−1^, pointing to a stronger interaction between the molecules under refrigeration conditions. This strength is lost with increasing temperature from 5 °C to 15 °C, 25 °C and 35 °C, by means of 15–20% reduction in the encapsulation constant every 10 °C (i.e., approximately a decrease of 1.8% per °C).

The thermodynamic parameters were established by Equations (6) and (7), revealing that the process was exothermic due to the negative enthalpy value (Δ*H°* = −14.12 ± 0.71 kJ/mol), which is common in this type of encapsulation due to the displacement of the water molecules from the inner cavity to allocate the stilbene. In contrast, the entropy value was positive (Δ*S°* = −29.51 ± 1.48 J/mol), which is not in agreement with previous results with analogues in which entropy was unfavourable, such as piceatannol, oxyresveratrol, or gnetol [23,24,25]. However, the less polar nature of this stilbene due to the presence of a methoxy group could be the reason, due to the release of structure hydration water to the bulk. This was also observed in the encapsulation of other phenolic compounds with HP-β-CD, such as caffeic acid and rosmarinic acid [26]. Finally, the negative Gibbs free energy value (25 °C Δ*G°* = −22.92 ± 1.15 kJ/mol) indicated that the reaction was spontaneous and was similar to that of the analogue piceatannol (25 °C Δ*G°* = −23.5 ± 1.2 kJ/mol [23]).

### 3.6. Enhancement of the Water Solubility of RHA with HP-β-CD

**Figure 5:**
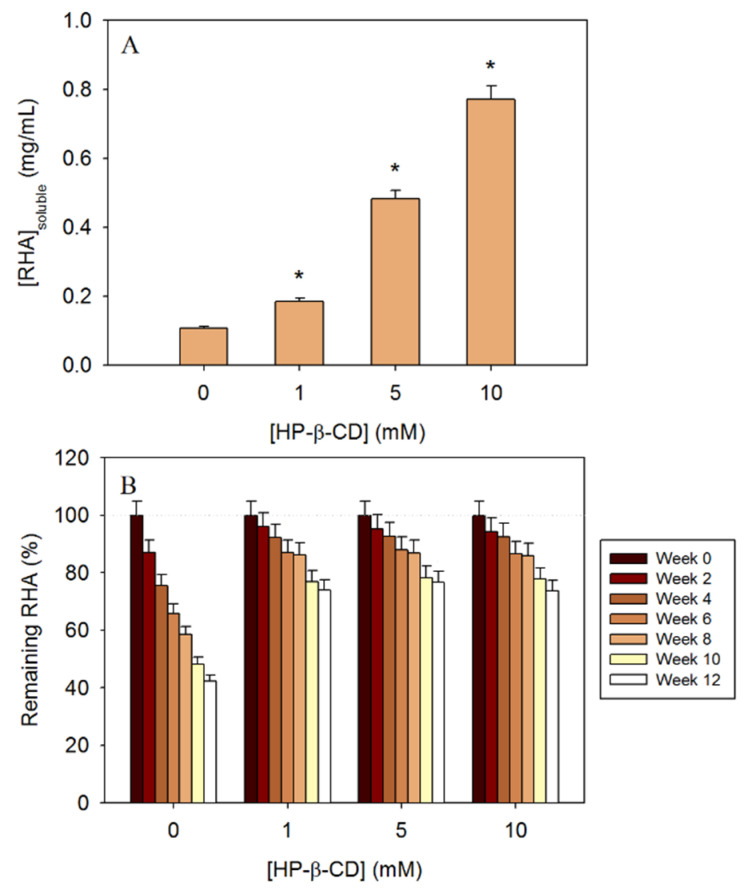
Effect of molecular encapsulation on the (**A**) water solubility and (**B**) shelf life of RHA (25 °C pH 7). * Significance *p* < 0.05.

The concentration of soluble RHA was calculated by considering its molar attenuation coefficient ε_326_ = 30,094 M^−1^ cm^−1^. The results showed that the methoxy group of RHA clearly affects the hydrophobicity of the molecule. While the aqueous solubility of piceatannol was previously established as 0.50 mg/mL [1], RHA solubility was five times lower, i.e., 0.11 mg/mL (Figure 5A). However, the use of CDs improved this value, formulations with 5 mM and 10 mM of HP-β-CD increased more than fourfold and sevenfold the amount of solubilised RHA, respectively. In fact, above 5 mM of HP-β-CD, RHA showed a similar or higher solubility than basal solubility of piceatannol. Moreover, even the lowest concentration of 1 mM was able to significantly increase the solubility by 71%, which favours the applications of this complex. This fact encourages the use of CDs together with RHA to avoid solubilisation problems in water-based products.

### 3.7. Stability Test of CD Inclusion Complexes with RHA

In the absence of encapsulating agents, RHA degraded linearly (R^2^ > 0.98) with a negative slope of almost −5 (Figure 5B). By the tenth week, the solution had already lost half of the stilbene, and, at the end of the assay, only 42% of the initial amount of RHA remained, compromising the long-term viability of an enriched product.

In contrast, in the presence of CDs, the average slope decreased to −2 (R^2^ > 0.97), with more than 73% of RHA remaining after 12 weeks of storage. Moreover, there was no significant difference between the concentrations so that only 1 mM is sufficient to improve the stability of the molecule. This new formulation seems more suitable than the use of free stilbenes if the final product is intended to be stored and exported over longer periods of time.

## 4. Conclusions

The antiproliferative effects of RHA against colorectal cancer cells were evaluated for the first time, revealing a similar or even better inhibition of cancer cell growth compared to resveratrol and piceatannol. To solve potential solubilisation problems in the development of functional foods, cosmetics, or drugs fortified with RHA, a novel formulation of RHA with CD was designed. Inclusion complexes with natural and modified CDs were characterised with a 1:1 stoichiometry. The highest affinity was observed for the HP-β-CD complexes, with an encapsulation constant *K_F_* = 10,308.89 ± 515.44 M^−1^ and docking score −10.2. Molecular docking revealed stabilization by hydrogen bonding. In addition, neutral pH and refrigeration temperature were the most suitable conditions for storage of the complexes. Above 1 mM of this CD, the RHA is fully complexed, and its stability at room temperature increased from 42% to more than 73% after 3 months of storage. Molecular encapsulation also improved the solubility of RHA by achieving a sevenfold increase at the highest concentration tested. Taken together, these results encourage the use of RHA as a bioactive ingredient in food, drugs, or cosmetics.

## Figures and Tables

**Table 1:** Correlation coefficients, encapsulation constants, and molecular docking scores obtained for RHA inclusion complexes with different CDs (25 °C pH7).

Cyclodextrin	R^2^		*K_F_* (M^−1^)	Score
	1:1 Model	1:2 Model		
α-CD	0.993	0.881	520.32 ± 26.02	−7.1
β-CD	0.942	0.844	2990.11 ± 149.51	−9.2
γ-CD	-	-	-	−6.5
HP-β-CD	0.995	0.895	10,308.89 ± 515.44	−10.2
M-β-CD	0.994	0.891	5189.11± 259.46	−9.2

## Data Availability

Not applicable.
